# Usual Dietary Intake Estimation Based on a Combination of Repeated 24-H Food Lists and a Food Frequency Questionnaire in the KORA FF4 Cross-Sectional Study

**DOI:** 10.3389/fnut.2019.00145

**Published:** 2019-09-06

**Authors:** Patricia Mitry, Nina Wawro, Julia Six-Merker, Dorothee Zoller, Carolin Jourdan, Christa Meisinger, Sigrid Thierry, Ute Nöthlings, Sven Knüppel, Heiner Boeing, Jakob Linseisen

**Affiliations:** ^1^Institute of Epidemiology II, Helmholtz Zentrum Munich, German Research Center for Environmental Health (GmbH), Munich, Germany; ^2^German Center for Diabetes Research (DZD e.V.), Düsseldorf, Germany; ^3^Chair of Epidemiology, Ludwig-Maximilians-Universität München, UNIKA-T, Augsburg, Germany; ^4^FGK Clinical Research GmbH, Munich, Germany; ^5^Department of Nutrition and Food Sciences, University of Bonn, Bonn, Germany; ^6^Department of Epidemiology, German Institute of Human Nutrition Potsdam-Rehbruecke (DIfE), Nuthetal, Germany; ^7^ZIEL Institute for Food and Health, Technical University of Munich, Freising, Germany

**Keywords:** dietary assessment, food list, food frequency questionnaire, KORA, epidemiology

## Abstract

**Background:** Estimation of usual dietary intake poses a challenge in epidemiological studies. We applied a blended approach that combines the strengths provided by repeated 24-h food lists (24HFLs) and a food frequency questionnaire (FFQ).

**Methods:** At least two web-based 24HFLs and one FFQ were completed by 821 participants in the KORA FF4 study. Consumption probabilities were estimated using logistic mixed models, adjusting for covariates and the FFQ data on consumption frequency. Intake amount of a consumed food item was predicted for each participant based on the results of the second Bavarian Food Consumption Survey (BVS II). By combining consumption probability and estimated consumption amount, the usual food intake for each participant was estimated. These results were compared to results obtained without considering FFQ information for consumption probability estimation, as well as to conventional FFQ data.

**Results:** The results of the blended approach for food group intake were often higher than the FFQ-based results. Intraclass correlation coefficients between both methods ranged between 0.21 and 0.86. Comparison of both methods resulted in weighted kappa values based on quintiles ranging from fair (0.34) to excellent agreement (0.84). Omission of FFQ information in the consumption probability models distinctly affected the results at the group level, though individual intake data were slightly affected, for the most part.

**Conclusions:** Usual dietary intake data based on the blended approach differs from the FFQ-based results both in absolute terms and in classification according to quintiles. The application of the blended approach has been demonstrated as a possible tool in nutritional epidemiology, as a comparison with published studies showed that the blended approach yields reasonable estimates. The inclusion of the FFQ information is valuable especially with regard to irregularly consumed foods. A validation study including biomarkers of dietary intake is warranted.

## Background

Nutritional epidemiology tries to clarify the role of diet in the context of health and disease in large populations. The valid assessment of dietary intake is key for identifying diet disease associations. However, dietary intake assessment methods are substantially affected by measurement errors and other challenges (1–3). As people often eat a variety of foods without knowing the exact ingredients, and as they tend to forget quickly what they consumed during the recent past, the assessment of dietary intake is challenging (4). Questions persist regarding the most appropriate instrument to measure dietary intake at the individual level in large cohort studies. Although food frequency questionnaires (FFQs) have long been the instrument of choice in epidemiologic studies, FFQs assesses dietary intake with both systematic and random error that may affect estimates of diet-disease associations (5–8). Several biomarker studies have cast doubt on whether the FFQ has sufficient precision to allow detection of moderate but important diet-disease associations (9–12). de Boer et al. (13) recommend applying 24 h dietary recalls (24 h-DR) on at least two non-consecutive days per participant in combination with a food propensity questionnaire in order to derive appropriate estimates to rank individuals according to their intakes. A 24 h dietary assessment tool that can be efficiently and cost-effectively administered over time is an attractive assessment method for capturing dietary information in large epidemiological studies (14). As the consumption of rarely consumed foods might be missed by collecting only a small number of recalls (15), including a FFQ improves the accuracy of the assessment (8, 13, 16–19).

In the context of a new cohort study, the German National Cohort, 24-h food lists (24HFL) that can be filled-in online in <10 min on average have been developed (16). By combining information from a conventional FFQ with 24HFL data, the calculation of an individual's usual dietary intake should be less affected by measurement error. Suitable two-part statistical models have been developed, which use dietary information from 24 h-DRs to estimate individuals' usual dietary intakes, while also including FFQ data as a covariate (20–24), e.g., the National Cancer Institute (NCI) method or the Multiple Source Method (MSM). The idea of the two-part statistical model to separate the estimation of consumption probability from the daily consumption amount was adapted here in a blended approach, which applies the simplified dietary assessment (24HFL) to estimate consumption probabilities, complemented by the use of an external study population to estimate the daily consumption amounts.

We applied both dietary assessment methods (repeated 24HFL and a FFQ) in the German population-based KORA (Cooperative Health Research in the Region of Augsburg) study. In this paper, we derived usual food intake estimates from our blended approach by combining information from 24HFLs data and an FFQ. We compare those estimates with results based on the FFQ alone. In addition, the relevance of the FFQ information as an adjustment variable in the models for estimating intake probability within this blended approach was investigated.

## Materials and Methods

### The KORA FF4 Study

KORA is a population-based research platform with subsequent follow-up studies in the fields of epidemiology, health economics, and health care research (25). The KORA S4 survey included 4,261 participants aged 25–74 years and was conducted from 1999 to 2001 in the region of Augsburg, Germany. The KORA S4 sample is representative for the Bavarian population. The KORA FF4 study is the second follow up examination of the S4 participants conducted in 2013/2014. A major aim of this study was to determine health status changes that occurred during the previous 14 years. The present analysis comprises a subset of 821 participants (425 men and 396 women) of KORA FF4. This subset of 821 participants completed at least two web-based 24HFLs and one FFQ before the end of November 2014. Information on sociodemographic variables and lifestyle factors was collected in an extensive standardized face-to-face interview at the study center. Furthermore, all participants underwent anthropometric measurements which included weight and height measurement. All KORA studies are approved by the Ethics Committee of the Bavarian Medical Association (Bayerische Landesärztekammer). Written informed consent was obtained from each participant in accordance with institutional requirements and the Declaration of Helsinki principles.

### Dietary Assessment

Two instruments were used in KORA FF4 in order to collect information on dietary intake, i.e., repeated 24HFLs and an FFQ. The 24HFL is a questionnaire which assesses foods consumed over the past day, while the food frequency questionnaire (FFQ) addresses the diet of the past 12 months. Both questionnaires were primarily offered as web-based forms. On request, paper forms were made available as well. However, the data presented here are confined to web-based data (and thus not affected by the handling of missing values).

The 24HFL is a closed and structured list of 246 food items for the identification of foods and drinks consumed over the past day. It neither assesses meals nor portion sizes. For each food item, either yes or no must be answered regarding consumption during the past 24 h. Freese et al. (16) describe that the 246 food items were chosen such that at least 75% of variation in nutrient intake was covered. It is important to note that this 24HFL is not a stand-alone dietary assessment instrument but is to be used in blended approaches as we have done here. Further details are described elsewhere (16).

The FFQ is based on the German version of the multilingual European Food Propensity Questionnaire (EFPQ) (26). Participants were asked to report the usual frequency of consumption and the usual portion size of the consumed food and drink items during the past 12 months. Participants used pictograms to estimate the portion size. The FFQ included 148 items. The frequency of food item consumption was assessed in specified categories (never, once a month or less, two or three times a month, one to two times a week, three to four times a week, five to six times a week, one time per day, two times per day, three times per day, and more than three times per day). Some questions were included on type of added fat, milk or sugar and the cooking method applied.

All participants filled out the first 24HFL during the first study center visit. Therefore, the first collection day of the 24HFL was a weekday. Within the following 3 months, participants were asked (per email or by phone) to complete two additional 24HFLs at home. Overall, two 24HFLs were to be completed on a work day (Monday–Friday) and the third on a weekend day (Saturday or Sunday). In fact, the true ratio of 5:2 from weekend days to weekend days may not be adequately captured by this, as the recall assesses the day before. For example, on a Monday, the consumption on a Sunday is reported. This shift can lead to a slight overrepresentation of weekend days. Furthermore, the participants were requested to complete an FFQ after the study center visit (before completing the second 24HFL). If the respondents did not fill out the questionnaire within 7 days, they were reminded by a phone call. If there was no reply more than 1 week after the first reminder, a second reminder was given. All 821 participants filled out at least two 24HFLs and 684 participants filled out a third 24HFL.

### Estimation of Usual Food Intake

We term our approach of estimating usual food intake by including recall data and FFQ information a “blended approach.” It follows the idea of the two-part statistical model of separation of consumption probability from consumption amount on consumption days, as proposed by the NCI and MSM methods (20–22, 24). Briefly, the two-part statistical model estimated intake probability and intake amount on a consumption day of a certain food via mixed models based on 24 h recall data. The usual food intake is then derived from those two parts, as described in detail below.

We applied a logistic linear mixed model (PROC NLMIXED) to estimate the consumption probability for each food item and for each participant based on the information provided by at least two 24HFLs assuming classical measurement error model (27). Models were adjusted for age, sex, BMI, smoking, physical activity level, education level, and the consumption frequency for each food item derived from the FFQ. For comparison, models without FFQ information as a covariate were also run.

As the 24HFL does not assess portion sizes, we predicted the consumption amount for each food item on a consumption day on the basis of the second Bavarian Food Consumption Survey (BVS II) (28). BVS II is a cross-sectional and population-based study conducted in 2002–2003. It was designed to investigate dietary and lifestyle habits of the Bavarian population. Within the BVS II study, dietary intake was assessed with three 24-h recalls using the software EPIC-Soft. In total, data of 2,708 recalls from 932 participants were available. By means of mixed linear models, the consumption amount of each food item was modeled with the BVS II data set. Models were adjusted for age, sex, BMI, smoking, physical activity, and education level. Due to skewed distributions, amounts have been transformed by Box-Cox transformations. Estimates were derived for each parameter in the model. For every individual in the KORA FF4 study, the consumption amount for each food item on a consumption day was predicted by using these parameter estimates. Estimated amounts were back-transformed to the original scale. Negative values were set to zero.

The estimated intake probability and the estimated consumption amount for each food item were multiplied, approximating each participant's usual food intake. This procedure is reasonable, as we do have predicted amounts of consumption, which can be interpreted as expected values of the individual amounts of consumption. Furthermore, the EPIC-Potsdam study showed that portion size adds little information to the variance in food intake using FFQ data, implying that consumption frequency has a stronger influence on the variation in food and nutrient intake between persons than portion size does (29). The model for the consumption probability as well as for the amount of consumption both incorporate the same covariates, thereby linking both modeling parts.

For the estimations based on the FFQ data only, the amount of dietary intake was calculated in grams per day for all food items from the FFQ, as described elsewhere in more detail (30).

For both methods, food items were combined into 16 food groups and 21 subgroups according to the EPIC-Soft classification scheme (31). Using food groups circumvents the problem of different food items being evaluated by different instruments. Hence, the combination of both dietary assessment instruments is possible.

### Definition of Covariates

Height and weight were measured to the nearest 0.1 cm and 0.1 kg, respectively. BMI was calculated as the ratio of weight by squared height (kg/m^2^). Underweight was defined as having a BMI < 18.5 kg/m^2^, normal weight 18.5–24.9 kg/m^2^, overweight 25–29.9 kg/m^2^, and obesity ≥30 kg/m^2^ (32). Physical activity was assessed on a four level graded scale by the amount of regular leisure time exercise per week during summer and winter. Based on those assessments, we used two levels of physical activity for adjustment in our analyses, i.e., physically inactive and physically active (33). The educational level in KORA was assessed on a two level scale, i.e., <12 years of education or 12 years of education or more.

### Statistical Analysis

The descriptive data are presented as mean and standard deviation for the continuous variables or as numbers and percentages for categorized variables. Food intake data in men and women were presented as mean, standard deviation, median, percentiles, and minimum and maximum for the blended approach including FFQ information. We compared intake estimates derived by the blended approach, including FFQ information with (i) estimates based on the conventional FFQ and (ii) estimates based on the blended approach without using FFQ information. Intraclass correlation coefficients and corresponding 95% confidence intervals were calculated (34). Values <0.5, between 0.5 and 0.75, between 0.75 and 0.9, and >0.90 are indicative of poor, moderate, good, and excellent comparability, respectively [see e.g., (35)]. Moreover, quintiles were defined based on gender-specific distributions of intake data to test the agreement in ranking participants regarding their food consumption as estimated by both methods. The proportion of participants classified into the same, adjacent, or opposite quintile for both methods was calculated. The agreement of individual quintile classification between both methods was evaluated and weighted Kappa values were calculated (36). The disagreements were weighted according to their squared distance from perfect agreement. The kappa statistic could not be derived for food subgroup “white meat.” Unless other food groups or subgroups were reported, the “white meat” food group was not combined with any other food group. For this reason, only five distinct values are available, representing the five different frequencies of consumption assessed by the FFQ. For other food groups or subgroups this is not limiting, as the range of possible values is extended by grouping the items. The Kappa statistic indicates excellent agreement for values over 0.80, good agreement for values between 0.61 and 0.80, moderate agreement for values between 0.41 and 0.60, fair agreement for values between 0.21 and 0.40, and poor agreement for values ≤0.20 (37).

All statistical analyses were performed with SAS, Version 9.3 (SAS Inc.).

## Results

The present study includes the 425 men and 396 women who exclusively completed web-based dietary questionnaires (Table 1). All participants filled out one FFQ. Six hundred and eighty-four participants completed three 24HFLs and one hundred thirty-seven participants completed two 24HFLs. The mean age of the participants at examination date was 54.1 ± 9.9 years. The mean BMI of the participants was 28.1 ± 4.9 kg/m^2^ in men and 26.0 ± 4.9 kg/m^2^ in women. Among men, 26.8% had a normal body weight, whereas 49.7% of women had a normal body weight. At time of assessment, most of the participants (86.4%) were non-smokers, only 13.6% of men and women smoked. Women were slightly more physically active than men. Table 1 also reports the characteristics of the entire cohort. As expected, the restriction to those participants that filled out all questionnaires online did not lead to a representative subsample. The subsample we investigated consisted of younger participants who were less overweight, more physically active, and slightly more educated than the cohort as a whole.

**Table 1:** Characteristics of all KORA FF4 participants and the subsample that completed dietary questionnaires online^*^.

		**Sub sample**				**Total sample**			
		**Men** **(*****n*** **=** **425) (51.8%)**		**Women** **(*****n*** **=** **396) (48.2%)**		**Men** **(*****n*** **=** **1,097) (48.3%)**		**Women** **(*****n*** **=** **1,175) (51.7%)**	
		***n***	**%**	***n***	**%**	***n***	**%**	***n***	**%**
Age (years)	<50	143	33.6	174	43.9	264	24.1	290	24.7
FF4 examination	50–59	128	30.1	135	34.1	252	23.0	309	26.3
	60–69	104	24.5	72	18.2	270	24.6	286	24.3
	≥70	50	11.8	15	3.8	311	28.4	290	24.7
BMI (kg/m^2^)	Normal-weight	114	26.8	197	49.7	249	22.7	446	38.0
	Pre-obese	198	46.6	126	31.8	529	48.2	411	35.0
	Obese	113	26.4	73	18.4	319	29.1	318	27.1
Current smoking	Smoker	58	13.6	54	13.6	182	16.6	168	14.3
	Non-smoker	367	86.4	342	86.4	915	83.4	1,007	85.7
Physically active	Yes	266	62.6	274	69.2	609	55.5	693	59.0
	No	159	37.4	122	30.8	488	45.5	482	41.0
Years of education	<12	256	60.2	250	63.1	767	69.9	927	78.9
	≥12	169	39.8	146	36.9	330	30.1	248	21.1

n, number of participants; Normal-weight, BMI <25 kg/m^2^; Pre-obese, BMI 25–29.99 kg/m^2^; Obese, BMI ≥ 30 kg/m^2^.

* *At least two out of three 24HFL and one FFQ*.

Table 2 gives descriptive data on dietary intake of each food group and subgroup in g/d as calculated by means of the blended approach (at least two 24HFL, with FFQ as a covariate), stratified by sex. The mean intake of vegetables, fruits, and dairy products was higher in women than in men, namely 202, 154, and 226 g/d, respectively, in women as opposed to 158, 142, and 179 g/d in men. In contrast, the average consumption of potatoes, cereals or cereal products, meat, and alcoholic beverages were clearly higher in men than in women. Men consumed 61, 200, 149, and 363 g/d, whereas women ate 49, 152, 90, and 80 g/d, respectively. The mean meat intake in men amounted to 149 g/d of which 44.6% was processed meat. In women, average meat intake was 90 g/d, whereof 37.8% was processed products.

**Table 2:** Distribution characteristics of usual dietary intake of food groups by blended approach including FFQ information.

**Food group, subgroup (g/day)**	**Men (*****n*** **=** **425)**					**Women (*****n*** **=** **396)**				
	**Mean**	**SD**	**Percentiles**			**Mean**	**SD**	**Percentiles**		
			**25**	**50**	**75**			**25**	**50**	**75**
Potatoes	61.0	18.7	48.0	56.9	70.0	49.1	16.6	37.8	45.1	56.4
All vegetables	157.6	50.1	122.4	148.6	186.3	201.8	70.0	149.9	190.4	240.3
Leafy vegetables	25.2	11.7	15.1	24.1	30.2	24.9	11.1	14.6	23.1	33.3
Fruiting vegetables	70.0	29.4	48.9	62.6	84.1	92.7	36.2	63.6	85.9	112.6
Root vegetables	18.9	14.9	10.3	14.3	21.7	34.8	30.6	16.2	24.7	40.5
Cabbage family	15.5	7.7	10.6	13.4	17.8	15.2	6.7	10.5	13.4	18.1
Legumes	5.4	3.7	3.2	4.2	6.2	6.9	4.2	4.2	5.5	8.3
Fruits	142.2	81.4	73.7	127.1	191.2	154.2	79.5	91.0	140.4	205.8
Nuts	7.9	8.7	3.2	4.5	9.1	7.3	7.8	2.6	4.2	9.2
Dairy products	179.2	102.2	102.0	155.9	233.8	226.4	104.8	144.6	212.2	291.4
Milk	81.8	79.7	16.9	56.9	122.8	106.8	77.1	42.6	91.7	157.9
Yogurt	40.0	43.2	11.6	21.5	51.7	54.0	44.7	17.2	39.6	76.1
Cheese	30.9	14.7	19.3	28.2	38.9	29.7	12.5	18.9	29.0	38.3
Cereals, cereal products	199.8	47.6	166.3	192.6	225.8	152.4	38.3	125.8	146.1	172.5
Bread	112.4	35.7	89.9	107.9	133.7	80.2	25.6	61.9	77.2	95.6
Pasta, rice, other grains	60.1	14.6	49.1	57.7	68.4	46.8	13.7	37.4	43.0	53.9
All meat and meat products	149.4	41.9	120.9	146.0	171.7	89.8	24.3	73.6	87.3	101.6
Red meat	58.6	15.8	47.9	55.2	66.8	36.5	8.3	30.7	34.9	41.3
White meat	17.1	8.4	11.4	13.5	20.8	14.3	7.8	9.1	11.7	16.7
Processed meat	66.7	30.4	45.9	62.2	79.4	33.9	15.6	23.9	30.8	40.2
Fish	23.2	15.0	12.8	18.3	28.8	18.4	10.7	10.7	14.8	23.9
Eggs	17.2	11.1	10.3	13.8	20.2	15.9	9.8	9.9	13.0	18.7
Added Fat	28.3	8.3	22.9	28.7	34.1	20.2	5.8	15.7	20.7	23.5
Sugar, confectionery	39.7	16.3	27.0	37.9	49.8	35.6	14.5	24.5	32.7	43.1
Cakes	56.0	22.3	38.7	50.8	68.3	47.1	15.4	35.9	44.3	55.5
Non-alcoholic beverages	1562.8	311.1	1369.3	1548.6	1726.0	1606.3	270.5	1415.5	1586.6	1794.6
Soft drinks	91.0	188.8	6.3	11.1	74.1	35.4	95.1	3.0	4.5	8.4
Alcoholic beverages	362.5	253.8	110.0	294.3	591.3	79.6	75.5	28.6	52.3	99.0
Beer	294.1	245.0	52.1	222.2	524.6	22.3	47.1	6.3	7.3	8.8
Wine	48.1	56.4	14.6	21.1	56.3	41.6	49.6	14.0	21.6	45.4
Condiments, sauces	25.8	6.8	21.1	24.6	29.1	22.6	6.0	18.5	21.2	25.4
Soups, bouillon	27.4	20.7	16.1	21.1	30.0	25.2	16.0	15.1	20.2	28.9

As shown in Table 3, the median food group intake obtained with the blended approach including FFQ information were often higher compared to the median intake derived from the conventional FFQ. For example, median differences of −35 g/d for vegetable intake, −33 g/d for fruits, −48 g/d for cereals or cereal products, and −25 g/d for total meat was observed in men. In women, the differences of medians between both methods were less uniform e.g., +12 g/d for potatoes, −34 g/d for vegetables and +345 g/day for non-alcoholic beverages. Intraclass correlation coefficient between data based on the blended approach and the FFQ based data ranged from 0.21 for legumes to 0.83 for eggs in men. In women values ranged from 0.26 for non-alcoholic beverages to 0.86 for eggs. Correlation coefficients below 0.5 were observed in men for all vegetables, leafy-, root vegetables, cabbage family, legumes, added fat, alcoholic-, non-alcoholic beverages, beer, and wine. Those low values were seen in women for the intake of all vegetables, leafy-, fruiting-, root vegetables, cabbage family, legumes, bread, red-meat added fat, alcoholic-, non-alcoholic beverages and condiments, and sauces. The weighted Kappa statistic was used to evaluate agreement between both methods in terms of attribution to the quintiles of the sex-specific intake distributions. The highest kappa values of 0.84 were calculated for yogurt in men and of 0.82 for eggs in women. Excellent agreement was observed in men for the intake of dairy products and yogurt, and in women for egg and cheese intake. Moderate agreement was found for the intake of the “all vegetables” group (0.5 in men, 0.51 in women) and for nearly all vegetables subgroups, too. Generally, good agreement was observed in most of the food groups and subgroups. Accordingly, the proportion of individuals attributed to the opposite quintiles was fairly low. For non-alcoholic beverages a Kappa value of 0.39 in men and 0.34 in women, as well as for cabbage intake in men (Kappa = 0.39) suggested fair agreement between both methods.

**Table 3:** Comparison of usual dietary food intake of food groups: blended approach including FFQ information vs. conventional FFQ.

**Food group, subgroup (g/day)**	**Men (*****n*** **=** **425)**							**Women (*****n*** **=** **396)**						
	Difference, median^*^	**ICC**	**95% CI**	**Weighted κ**	**Same quintile (%)**	**Adjacent quintile (%)**	**Opposite quintile (%)**	Difference, median^*^	**ICC**	**95% CI**	**Weighted κ**	**Same quintile (%)**	**Adjacent quintile (%)**	**Opposite quintile (%)**
Potatoes	6.1	0.60	(0.54; 0.66)	0.68	41.2	41.6	0.0	11.6	0.53	(0.45; 0.59)	0.71	47.7	37.1	0.0
All vegetables	−35.4	0.43	(0.35; 0.51)	0.50	35.8	36.0	0.9	−33.5	0.41	(0.33; 0.49)	0.51	35.9	36.9	1.5
Leafy vegetables	−5.8	0.43	(0.35; 0.50)	0.48	36.0	32.5	1.4	−3.4	0.37	(0.28; 0.45)	0.46	34.8	34.1	0.8
Fruiting vegetables	−17.6	0.55	(0.48; 0.61)	0.49	32.9	38.4	0.9	−12.6	0.49	(0.41; 0.56)	0.48	34.6	37.9	2.3
Root vegetables	−5.1	0.46	(0.38; 0.53)	0.46	32.9	36.7	1.6	−7.8	0.38	(0.29; 0.46)	0.50	34.8	35.9	1.8
Cabbage family	1.3	0.31	(0.22; 0.39)	0.40	33.4	33.6	2.4	6.7	0.27	(0.18; 0.36)	0.44	32.3	39.4	2.8
Legumes	−0.1	0.21	(0.11; 0.29)	0.41	30.1	36.7	2.1	−0.6	0.39	(0.31; 0.47)	0.43	35.4	34.8	2.5
Fruits	−32.9	0.67	(0.62; 0.72)	0.69	41.4	41.2	0.0	5.6	0.68	(0.62; 0.73)	0.65	37.6	42.9	0.3
Nuts	−2.3	0.77	(0.72; 0.80)	0.68	42.4	40.9	0.5	−1.3	0.83	(0.80; 0.86)	0.71	42.9	44.7	0.5
Dairy products	−10.3	0.77	(0.73; 0,81)	0.81	51.5	40.5	0.0	4.1	0.73	(0.69; 0.78)	0.79	50.5	39.4	0.0
Milk	−11.5	0.75	(0.71; 0.79)	0.78	46.6	42.6	0.0	−6.9	0.70	(0.64; 0.75)	0.75	45.2	43.9	0.3
Yogurt	1.2	0.74	(0.70; 0.78)	0.84	56.0	38.4	0.0	8.7	0.71	(0.65; 0.75)	0.77	49.7	38.6	0.0
Cheese	−0.9	0.79	(0.75; 0.82)	0.74	44.9	41.2	0.2	6.0	0.73	(0.68; 0.77)	0.81	49.7	42.4	0.0
Cereals, cereal products	−47.7	0.62	(0.56; 0.67)	0.66	40.5	42.8	0.5	−3.0	0.52	(0.45; 0.59)	0.64	40.2	41.4	0.5
Bread	−19.2	0.55	(0.49; 0.62)	0.61	41.6	37.2	1.6	2.2	0.44	(0.36; 0.52)	0.55	40.2	35.4	1.0
Pasta, rice, other grains	−25.1	0.68	(0.63; 0.73)	0.70	44.2	38.1	0.0	−6.5	0.62	(0.55; 0.68)	0.76	47.5	40.4	0.0
All meat and meat products	−25.4	0.74	(0.70; 0.78)	0.75	48.2	39.3	0.2	7.7	0.60	(0.53; 0.66)	0.77	52.5	36.4	0.3
Red meat	−14.8	0.69	(0.64; 0.74)	0.69	39.8	44.5	0.2	−3.7	0.45	(0.37; 0.53)	0.67	39.6	40.9	0.0
White meat	−0.9	0.76	(0.72; 0.80)	.	57.6	39.3	0.2	2.0	0.69	(0.64; 0.74)	.	53.0	43.2	0.3
Processed meat	−10.9	0.76	(0.72; 0.80)	0.72	44.9	40.7	0.2	5.9	0.66	(0.60; 0.71)	0.72	47.0	39.6	0.3
Fish	−0.6	0.77	(0.73; 0.81)	0.74	45.6	43.1	0.2	1.3	0.71	(0.66; 0.76)	0.76	48.5	38.6	0.0
Eggs	−3.0	0.83	(0.80; 0.86)	0.78	45.4	44.2	0.0	−2.6	0.86	(0.84; 0.89)	0.82	50.3	43.4	0.0
Added fat	−1.2	0.39	(0.30; 0.47)	0.51	34.6	38.4	1.6	5.1	0.29	(0.20; 0.38)	0.43	32.6	32.8	0.8
Sugar, confectionery	−3.5	0.59	(0.52; 0.65)	0.65	36.7	46.1	0.9	0.3	0.61	(0.54; 0.67)	0.70	46.7	35.9	0.3
Cakes	−9.6	0.65	(0.60; 0.71)	0.65	36.7	44.7	0.5	−5.4	0.54	(0.47; 0.61)	0.66	37.4	43.7	0.0
Non–alcoholic beverages	29.6	0.33	(0.25; 0.41)	0.39	31.1	38.4	4.0	344.7	0.26	(0.17; 0.35)	0.34	25.3	39.1	2.5
Soft drinks	−3.7	0.78	(0.74; 0.82)	0.67	43.8	36.2	0.0	−1.9	0.62	(0.55; 0.68)	.	35.6	35.6	1.0
Alcoholic beverages	55.8	0.34	(0.26; 0.43)	0.76	47.8	40.2	0.2	33.8	0.43	(0.34; 0.50)	0.68	42.2	40.4	0.5
Beer	5.0	0.33	(0.25; 0.41)	0.76	49.4	38.6	0.5	−1.3	0.34	(0.25; 0.42)	0.50	35.1	34.6	1.0
Wine	19.6	0.46	(0.38; 0.53)	0.67	40.9	40.2	0.2	16.5	0.52	(0.44; 0.59)	0.62	38.6	39.4	0.5
Condiments, sauces	3.4	0.47	(0.47; 0.61)	0.65	40.9	40.2	0.5	9.0	0.46	(0.37; 0.53)	0.68	41.2	43.9	0.5
Soups, bouillon	2.0	0.69	(0.63; 0.73)	0.58	37.2	38.8	0.5	7.5	0.62	(0.55; 0.67)	0.64	42.2	36.1	0.0

*Difference FFQ-based median—blended approach-based median.

Comparing the usual food intake estimates derived with and without including the FFQ information in the blended approach, we see that the median dietary intake of food groups and subgroups differed slightly between both methods (Table 4). The blended approach without FFQ information included yielded mostly higher usual food intake estimates. In men, a median difference of −2 g/d in meat intake, 4 g/d in fruit intake, 2 g/d in vegetable intake, 6 g/d in bread intake, and 22 g/d in alcoholic beverage consumption was observed. In women, a median difference of 2 g/d in potato intake, 5 g/d in vegetable intake, 4 g/d in legume intake, and −2 g/d in alcoholic beverage consumption was observed. The intraclass correlation coefficients ranged from 0.55 for white meat to 0.98 for beer in men, and from 0.58 for white meat to 0.98 for beer in women. For most food groups, the weighted kappa ranged above 0.8 in men and in women, indicating an excellent concordance between results based on the 24HFLwith and without using the FFQ information. Further, the percentages for the classification into opposite quintiles were <0.9% in all food groups and subgroups in men and women.

**Table 4:** Comparison of usual food intake estimated by the blended approach, including vs. not including FFQ information.

**Food group/subgroup (g/day)**	**Men (*****n*** **=** **425)**							**Women (*****n*** **=** **396)**						
	Difference median^*^	**ICC**	**95% CI**	**Weighted κ**	**Same quintile (%)**	**Adjacent quintile (%)**	**Opposite quintile (%)**	Difference median ^*^	**ICC**	**95% CI**	**Weighted κ**	**Same quintile (%)**	**Adjacent quintile (%)**	**Opposite quintile (%)**
Potatoes	1.5	0.74	(0.69; 0.78)	0.76	43.5	46.1	0.0	2.4	0.76	(0.72; 0.80)	0.77	44.4	46.5	0.3
All vegetables	2.1	0.93	(0.92; 0.95)	0.93	75.5	23.3	0.0	5.4	0.93	(0.91; 0.94)	0.90	71.7	25.5	0.0
Leafy vegetables	0.7	0.95	(0.94; 0.96)	0.90	69.6	28.2	0.0	0.9	0.94	(0.93; 0.95)	0.90	68.4	30.1	0.0
Fruiting vegetables	1.4	0.95	(0.94; 0.96)	0.94	80.7	18.4	0.0	2.5	0.92	(0.90; 0.93)	0.90	71.2	26.8	0.0
Root vegetables	0.4	0.93	(0.91; 0.94)	0.91	73.2	25.2	0.2	1.4	0.94	(0.93; 0.95)	0.91	71.7	26.3	0.0
Cabbage family	0.4	0.93	(0.92; 0.94)	0.90	74.6	23.1	0.5	0.5	0.88	(0.85; 0.90)	0.87	64.1	33.1	0.3
Legumes	0.2	0.96	(0.95; 0.96)	0.88	68.0	29.6	0.5	0.3	0.93	(0.92; 0.94)	0.90	69.9	26.8	0.0
Fruits	4.2	0.94	(0.93; 0.95)	0.93	76.9	22.1	0.0	3.6	0.92	(0.90; 093)	0.92	69.9	29.5	0.0
Nuts	0.3	0.87	(0.85; 0.89)	0.81	55.8	36.9	0.0	0.4	0.84	(0.81; 0.87)	0.85	62.4	33.6	0.3
Dairy products	1.6	0.93	(0.92; 0.94)	0.90	68.9	28.9	0.0	2.5	0.91	(0.89; 0.92)	0.89	66.2	31.1	0.0
Milk	2.5	0.95	(0.94; 0.96)	0.92	74.8	24.0	0.2	3.2	0.95	(0.94; 0.96)	0.93	74.7	24.7	0.0
Yogurt	0.7	0.91	(0.89; 0.92)	0.85	57.6	36.7	0.0	2.6	0.84	(0.81; 0.87)	0.86	61.1	35.6	0.3
Cheese	0.1	0.87	(0.84; 0.89)	0.85	60.7	33.2	0.2	0.5	0.82	(0.78; 0.85)	0.82	53.5	40.2	0.3
Cereals, cereal products	3.5	0.87	(0.84; 0.89)	0.84	56.9	37.6	0.0	0.4	0.86	(0.83; 0.88)	0.85	57.3	38.1	0.0
Bread	6.0	0.83	(0.80; 0.86)	0.85	59.5	35.8	0.0	3.1	0.84	(0.81; 0.87)	0.87	64.4	31.1	0.3
Pasta, rice, other grains	2.3	0.77	(0.72; 0.80)	0.76	49.4	38.6	0.2	2.5	0.72	(0.67; 0.76)	0.76	46.2	41.2	0.0
All meat and meat products	−1.6	0.77	(0.73; 0.81)	0.78	51.5	37.2	0.0	−0.3	0.74	(0.69; 0.78)	0.75	48.0	38.9	0.3
Red meat	0.4	0.68	(0.62; 0.72)	0.71	41.9	43.8	0.2	1.1	0.66	(0.60; 0;71)	0.70	41.4	42.2	0.3
White meat	1.3	0.55	(0.49; 0.62)	0.55	35.8	38.4	0.5	1.2	0.58	(0.51; 0.64)	0.57	32.8	42.9	0.3
Processed meat	1.6	0.81	(0.78; 0.84)	0.84	55.3	38.6	0.0	0.2	0.77	(0.73; 0.81)	0.83	54.5	39.9	0.0
Fish	1.7	0.80	(0.77; 0.83)	0.71	45.6	38.6	0.0	1.2	0.75	(0.70; 0.79)	0.67	41.9	40.2	0.5
Eggs	0.2	0.71	(0.66; 0.75)	0.70	44.9	38.8	0.5	0.5	0.65	(0.59; 0.71)	0.70	42.7	40.4	0.3
Added fat	0.4	0.91	(0.89; 0.93)	0.89	63.8	33.2	0.0	0.3	0.88	(0.86; 0.90)	0.86	61.6	34.3	0.3
Sugar, confectionery	0.9	0.91	(0.90; 0.93)	0.88	63.5	33.6	0.0	0.9	0.86	(0.83; 0.88)	0.86	56.3	40.2	0.0
Cakes	2.5	0.78	(0.74; 0.82)	0.86	57.6	38.1	0.0	1.8	0.80	(0.76; 0.83)	0.80	53.3	38.1	0.0
Non-alcoholic beverages	8.6	0.95	(0.94; 0.96)	0.94	76.2	23.3	0.0	8.0	0.94	(0.92; 0.95)	0.92	73.2	24.7	0.0
Soft drinks	−0.5	0.94	(0.92; 0.95)	0.97	88.0	11.5	0.0	−1.1	0.96	(0.95; 0.96)	0.95	81.3	18.2	0.0
Alcoholic beverages	22.4	0.97	(0.96; 0.97)	0.95	80.9	18.8	0.0	−2.4	0.96	(0.95; 0.96)	0.94	75.5	24.2	0.0
Beer	21.1	0.98	(0.97; 0.98)	0.94	75.8	24.2	0.0	−3.0	0.98	(0.98; 0.99)	0.43	35.1	32.3	0.8
Wine	1.4	0.87	(0.85; 0.89)	0.88	70.8	25.9	0.5	−0.1	0.92	(0.92; 0.93)	0.92	75.3	22.5	0.0
Condiments, sauces	0.0	0.80	(0.76; 0.83)	0.78	50.4	39.5	0.2	0.3	0.76	(0.72; 0.80)	0.74	49.0	37.9	0.0
Soups, bouillon	1.5	0.72	(0.68; 0.77)	0.76	47.3	43.8	0.9	0.9	0.62	(0.55; 0.68)	0.68	43.2	43.7	1.3

* *Mean (median) difference in the results of the blended approach: results without FFQ data as covariates—with FFQ data as covariates*.

**Figure 1:**
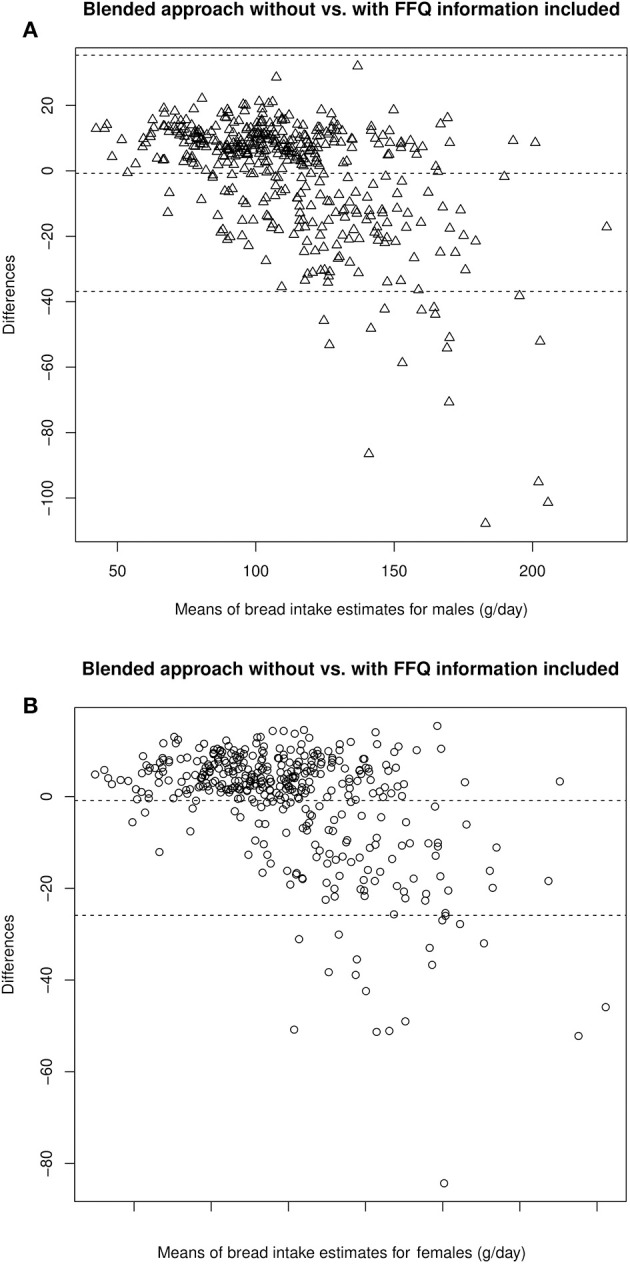
Bland-Altman Plots comparing estimates of bread intake by the blended approach with and without FFQ information in male **(A)** and female **(B)** participants.

The Bland-Altman plot for the food group “bread” in Figure 1 depicts the comparison of the blended approaches including and not including additional FFQ information on the individual level (y-axis) compared to their mean (x-axis). This food group showed, apart from the beverages, the highest median differences among men. We see an increasing deviation between both blended approaches with an increasing amount of consumption. These relatively large negative deviations occur when the estimate without FFQ information is much smaller than the estimate including FFQ information. This is, for example, the case when a certain food item was not consumed on the recall days but usually this food is consumed in notable amounts. Here, the FFQ correctly increases the estimate of the usual food intake. Most individual deviations are slightly positive, showing an overall good accordance of estimates on the individual level.

**Figure 2:**
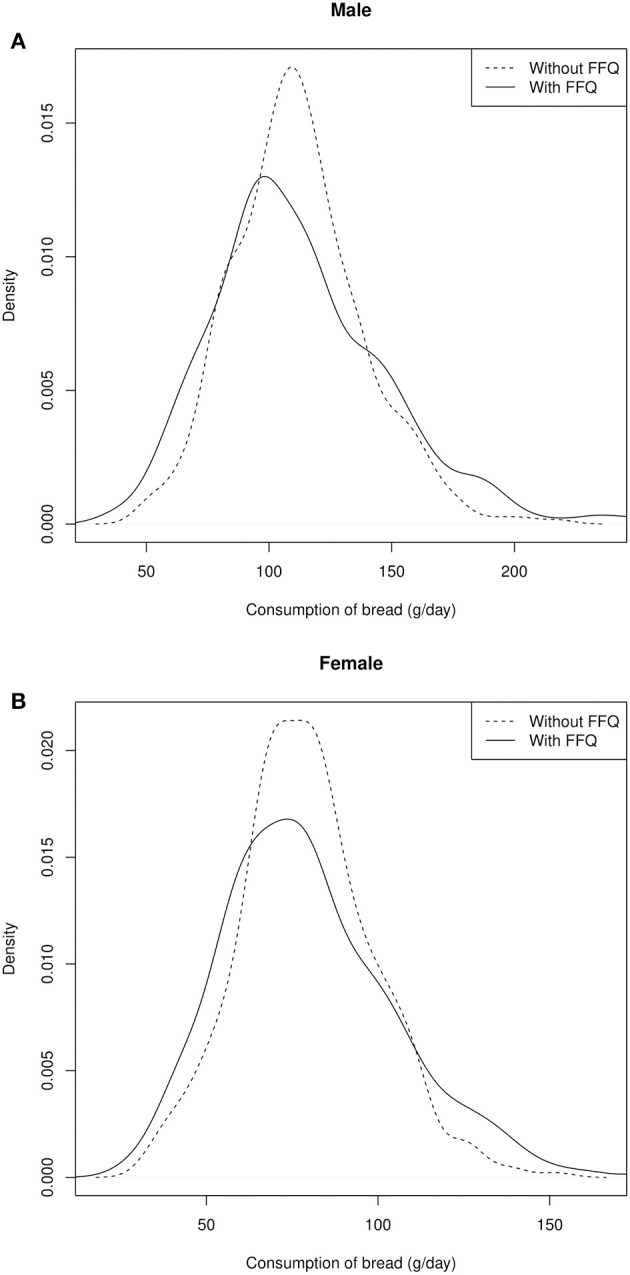
Distribution of the individual intake data of bread in male **(A)** and female **(B)** participants.

**Figure 3:**
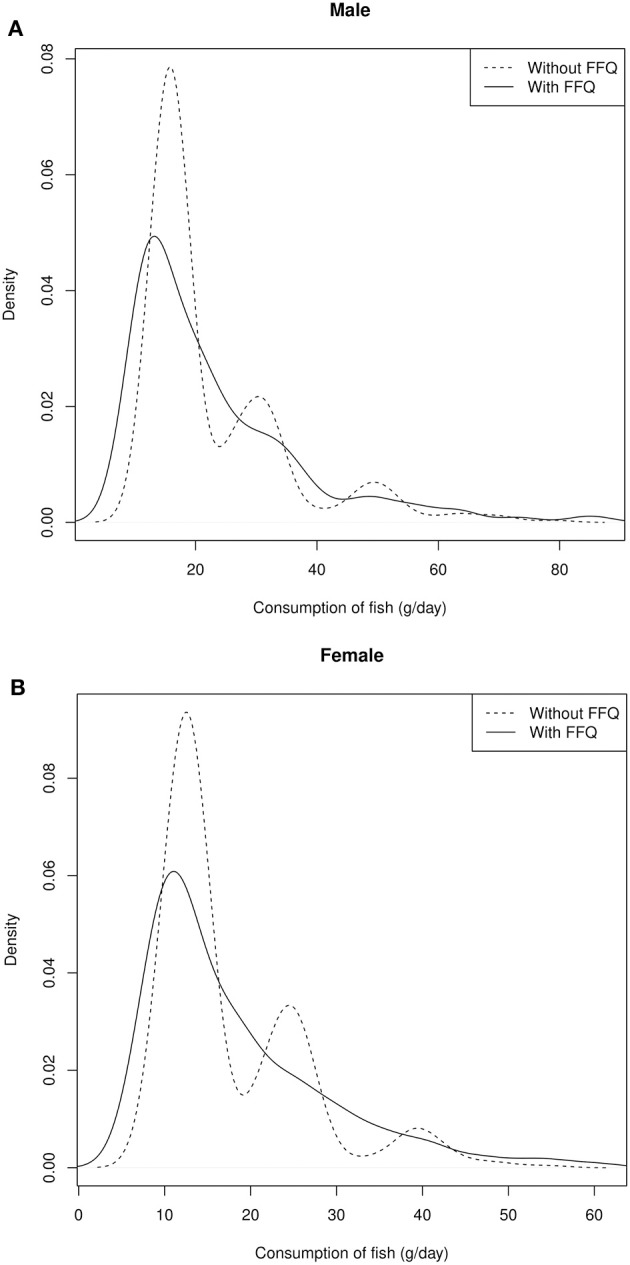
Distribution of the individual intake data of fish in male **(A)** and female **(B)** participants.

Although the median differences are small, inclusion of the FFQ information affects most intake distributions: On the group level, amplitudes of uni- and multimodal distributions of the individual values are cushioned and smoothed, resulting in slightly broader distributions. As two examples, the intake distribution of bread and fish in men and women are depicted in Figures 2, 3.

## Discussion

The aim of this study was to estimate usual dietary intake of the study participants based on at least two 24HFLs and one FFQ. Compared to the conventional FFQ-based intake calculation, the results of the blended approach including the FFQ information demonstrates distinct differences at the group level as well as at the individual level. Deviations in both directions were found, i.e., a higher or a lower usual food intake of food groups and subgroups. However, omission of FFQ information as adjustment variable in the estimation of the consumption probability led to distinct alterations in food group and subgroup intake data at the group level, especially regarding the tailes of the intake distributions. At the individual level, inclusion of the FFQ information did not lead to alterations for the majority of participants.

For a long time, the FFQ has been one of the most frequently used dietary assessment tool in large epidemiologic cohort studies, although it suffers from imprecision (3). Valid and precise dietary intake data at the individual level is the key to detecting moderate diet-disease associations, which may otherwise not be seen (38, 39). However, FFQ-based estimation of dietary intake relies on information about the usual frequency of food item consumption and standard portion sizes. Although inter-individual variation in portion sizes is lower than inter-individual variation in food intake (29), the imprecision in reporting usual food consumption frequency is not to be neglected. Using the information of repeated 24 h dietary recalls completed on non-consecutive days, the consumption probability of a food item per day can be estimated more accurately (24), especially concerning rarely consumed foods. It should be acknowledged that the FFQ was originally designed to rank participants according to their level of food consumption, rather than to give a precise estimate of food intake (40). In contrast to the FFQ, which relies upon the respondent's ability to quantify regular (mixed) food item consumption, dietary assessment of the consumed foods on the past day by the 24HFL is more simple, as the respondent chooses between a “yes” or “no” answer, depending on his or her previous 24-h dietary history. The 24HFL cannot be applied as a stand-alone instrument to assess dietary intake, but rather needs to be applied within a blended approach. Freese et al. (16) state that compared to the conventional 24HDR, the 24HFL allows quick and potentially frequent applications and can be used to estimate the probability of consumption. Further, the EPIC-Potsdam study showed that portion size adds little information to the variance of food intake using FFQ data, implying that consumption frequencies have a stronger influence on the variation in food and nutrient intake between persons than portion sizes do (29). The 24HFL is a tool designed to assess nutrition in a resource saving manner in a large study. We assume that the bias does not differ much from the application of classical 24HDR, which is a field of further research.

The blended approach we followed can give reasonable estimates for food items and food groups that are regularly consumed. Estimation of the usual food intake of rarely consumed food items is enhanced by additionally using the FFQ information as an adjustment variable in the two-part statistical models (20–22, 24).

Our results showed that the blended approach vs. conventional FFQ performed reasonably well in terms of reproducibility. The results of this study showed that the majority of the food groups had moderate to good ICC values. Only the food groups “vegetables,” “added fat,” “alcoholic beverages,” and “condiments and sauces” in men and women and their subgroups had ICC <0.55. Low ICC may indicate large within person variation and low precision of estimates [see e.g., (41)]. To obtain a realistic estimate of rarely consumed foods, we used the corresponding FFQ information as a covariate in the models.

The FFQ data is part of the blended approach. Thus, correlated errors of both estimation procedures are a consequence. However, distinct deviations between both methods were noted. Since we have not conducted a validation study to investigate the relative validity of the new assessment and calculation method, we can only compare our results to the available literature to assess their plausibility. Table 5 shows the comparison of the KORA FF4 usual food intake data that was derived with the blended approach including FFQ information to the results of previous studies in Germany that are based on repeated administration of 24 h dietary recalls. We compared our data to the National Food Consumption Survey II (NVS II) (42), the Bavarian Food Consumption Survey II (BVS II) (28), and the European Prospective Investigation into Cancer and Nutrition (EPIC-Potsdam) calibration study (24). The NVS II and BVS II both reported the (weighted) arithmetic means of two or three 24 h dietary recalls and EPIC-Potsdam utilized the ‘Multiple Source Method' for dietary intake estimations. All studies used the EPIC-SOFT software for standardized assessment of 24 h dietary recalls. The comparisons based on food groups circumvent any differences arising from different food items that were evaluated across the studies.

**Table 5:** Comparison of our blended approach results with other published German dietary intake studies.

	**Men**			**Women**			**Total**	
	**KORA FF4 (*n* = 425)**	**BVS II^**a**^ (*n* = 114)**	**NVSII^**b**^ (*n* = 4,571)**	**KORA FF4 (*n* = 396)**	**BVS II^**a**^ (*n* = 140)**	**NVSII^**b**^ (*n* = 1,740)**	**KORA FF4 (*n* = 821)**	**EPIC Potsdam^**c**^ (*n* = 393)**
	**Mean (sd) [g/day]**	**Mean [g/day]**	**Mean [g/day]**	**Mean (sd) [g/day]**	**Mean [g/day]**	**Mean [g/day]**	**Mean (sd) [g/day]**	**Mean [g/day]**
Potatoes	61 (18.7)	73	74	49 (16.6)	68	60	55 (18.6)	79
All vegetables	158 (50.1)	142	135	202 (70.0)	135	140	179 (64.4)	177
Fruits and nuts	154 (86.2)	143	178	168 (86.1)	190	225	161 (86.4)	272
Dairy products	179 (102.2)	162	171	226 (104.8)	220	190	202 (106.1)	222
Cereals, cereal products	200 (47.6)	274^d^	267^d^	152 (38.3)	219^d^	213^d^	177 (49.4)	199
All meat and meat products	149 (41.9)	170	158	90(24.3)	91	87	121 (45.6)	113
Fish	23 (15.1)	21	24	18 (10.7)	19	20	21 (13.3)	28
Eggs	17 (11.1)	11	12	16 (9.8)	9	12	17 (10.5)	16
Added fat	28 (8.3)	25	30	20 (5.8)	21	18	24 (8.3)	35
Sugar/Confectionery	40 (16.3)	41	67^e^	36 (14.5)	30	58^e^	38 (15.6)	58
Cakes	56 (22.3)			47 (15.4)			52 (19.8)	62
Non-alcoholic beverages	1,563 (311.1)	1,897	1,909	1,606 (270.5)	1,900	2,168	1,584 (292.9)	1,910
Alcoholic beverages	363 (253.9)	486	430	80 (75.5)	80	114	226 (236.8)	270

a Calculated on the basis of three 24 h dietary recalls (using EPIC-SOFT), age group 51–64 years (BVS Abschlussbericht 2003).

b Calculated on the basis of two 24 h dietary recalls (using EPIC-SOFT), age group 51–65 years (NVS II DGE 2012).

c EPIC Potsdam calibration study. Calculation based on two 24 h dietary recalls and one FFQ, mean age 57 years (24).

d Including cakes and sweet pastries.

e *Including drinking powder and drinking granulates*.

In 2004, the EPIC-Potsdam calibration study was conducted comprising 393 participants who have completed two 24 h dietary recalls and a FFQ within 1 year (24). Due to the use of a related calculation method, the EPIC-Potsdam data is best comparable with the KORA FF4 data regarding usual dietary intake. The National Food Consumption Survey II (NVS II) with 19,329 participants (age 14–80 years) recruited between November 2005 and January 2007, provided representative nutritional data for the German-speaking population across Germany. The reported results in this study were based on 13,753 respondents who had completed two telephone-administered 24 h dietary recalls (42). For the dietary intake estimation, the arithmetic mean of two 24 h dietary recalls for each food group was calculated. In 2002/2003, the cross-sectional BVS II study was conducted to investigate dietary and lifestyle habits of the Bavarian population. A sample of 1,050 Bavarian residents aged 13–80 years participated in a computer-assisted personal interview and completed three 24 h dietary recalls by telephone interview using EPIC-SOFT. The dietary intake estimate was derived as the arithmetic mean of three 24 h dietary recalls for each food group (28).

Overall, the average dietary intake data matched well between KORA FF4 and the EPIC-Potsdam calibration study for many reported food groups. Differences were greatest for the intake of potatoes, fruits and nuts, added fat and non-alcoholic beverages with lower mean intake data in the KORA FF4 study. When comparing the different intake estimates of KORA FF4 usual food intake with the results of the BVS II and the NVS II study, other food groups also had remarkable differences in mean intake data (vegetables, dairy products, cereals/cereal products). To some extent the observed dietary changes may be attributed to real changes of dietary habits over time, e.g., a decreasing trend in potato and bread intake and an increasing trend in vegetable, yogurt, and cheese consumption in Germany (42). Another reason could be that in KORA higher educated participants with a healthier diet took part.

As expected, the pure FFQ-based results for the KORA FF4 participants fit well with the results of a previous dietary analysis in a Southern German population (EPIC-Heidelberg) using a very similar FFQ (results not shown) (43).

In a recent study, Mitry et al. (44) examined the association between habitual meat intake and biomarkers in the BVS II study. Plasma concentrations of anserine, carnosine and pi-methylhistidine were assessed, They reported that these biomarkers can be utilized as biomarkers of habitual meat intake in epidemiologic studies. A further study in KORA FF4 is currently being performed in order to examine the correlation between dietary intake and fecal concentration of cholesterol and bile acids, with the aim of providing valid biomarkers of dietary intake.

Comparing the results of the blended approach and the conventional FFQ method for the estimation of usual dietary intake, values below 0.5 for the correlation coefficients indicated a larger discrepancy in the usual food intake estimates derived. Respondents were also grouped into quintiles for each food group to assess the agreement in ranking participants regarding their usual food consumption as estimated by both methods. On average, about 40% of men and women were similarly classified by both methods, and approximately another 40% were grouped in adjacent quintiles showing an overall good to moderate classification agreement of the participants across all food groups. The percentage of participants correctly classified into the same, adjacent, or opposite quintiles are comparable to those described by Bohlscheid-Thomas et al. (30). They reported the results of the validation study comparing the FFQ developed for the German part of EPIC vs. repeated 24 h dietary recalls. They showed that approximately 33% of participants were correctly classified into the same quintile and 70% were classified within the same or adjacent quintile (30). This also lends credit to a more reasonable estimation of food consumption by the blended approach as compared to conventional FFQ-based estimates.

In our study, the blended approach resulted in higher individual intake estimates as compared to the conventional FFQ. The median differences in food intake shown in Table 3 for men were mostly negative, i.e., lower estimates by the conventional FFQ, except for potatoes, cabbage family, yogurt, condiments, soups, and alcoholic and non-alcoholic beverages. Comparing the food group results with models not taking FFQ information into account shows only small deviations when looking at summary estimates e.g., the median or the categorization into sex-specific quintiles. However, the distributions of usual food intake data are slightly wider and smoother.

### Strength and Weaknesses of Our Study

Statistical methods are described in the literature to estimate the usual food intake of food items based on repeated 24 h dietary recalls with and without consideration of FFQ information. The idea of the two-part statistical model to separate the estimation of consumption probability from the daily consumption amount was adapted here in a blended approach, with the addition of portion size calculation using data from another local study. As the 24HFL did not assess portion sizes of the food items consumed, the amount of each consumed food item was predicted for each participant from the exact 24 h dietary recall data (using EPIC-SOFT) of the Bavarian Food Consumption Survey II (BVS II) (28), a cross-sectional population-based study in Bavaria, Germany. We took into account the most important determinants of consumption amounts as described elsewhere (45). One may argue that by using the BVS II study, we base our predictions on 10 years old data and this could bias the results. On the contrary, Table 5 shows that the main food groups mean estimates from the BVS II, conducted in 2003, and the National Food Consumption Study (NVS II), conducted in 2012, are mostly in good accordance. Hence, we do not expect strong influence from the 10 years difference. Nonetheless, the lack of an appropriate study population that is used to estimate intake amounts as we did with the BVS II can be seen as a limitation when transferring this idea to another context.

We are well aware that combining the two estimates, namely for the amount and the probability of consumption, affects the variance of the usual intake estimate. To assess the impact within our blended approach, a validation study including biomarkers like the doubly labeled water method and urinary nitrogen excretion is required.

The 24HFL is a closed list of food items, analogous to the FFQ. Although the combination of both instruments in a blended approach provides more information, very rarely consumed foods might still be missed by the closed lists.

Our study was based on those 821 participants of the KORA FF4 study who completed an FFQ and at least two web-based 24HFLs. Over 80% completed a total of three 24HFLs. Simultaneous model fitting for the probability of consumption for participants with either two or three recalls available makes use of all data available. We assume that it is unlikely that those filling in only two recalls differ substantially in their consumption frequencies from those who completed three 24HFL. The web-based administration ensured that no missing values existed that would have required additional assumptions and thus have introduced another source of variability. However, it is possible that more health-conscious persons were willing to fill out the questionnaires online (about 60% of the population), which may represent a selection of the population and thus limit the comparability of the results with that of other population-based studies. Misreporting was not considered in this study, i.e., no miss-reporters or outliers were excluded from the data set.

In the KORA FF4 study, data collection was done over a full year; however, data collection for the individual participants was completed within 3 months after study center visit. Thus, at the group level but not necessarily at the individual level, possible seasonal variation is captured. However, seasonal variation in the German diet is not very strong and may be limited to specific food items, especially fruit and vegetables (46). Dietary intake data at the food group or subgroup level are not expected to be distinctly affected by seasonal variation.

## Conclusions

Our paper compared the usual dietary intake estimates derived by a blended approach including FFQ information with those based on the FFQ alone. In addition, the relevance of the FFQ information used as an adjustment variable in the models to estimate intake probability within the blended approach was investigated in detail. A comparison with published studies showed that the blended approach yields reasonable estimates. On the group level, the inclusion of the FFQ information is valuable, especially with regard to irregularly consumed foods. However, a validation study including biomarkers of dietary intake is warranted.

## Ethics Statement

The KORA study protocols were approved by the Ethics Committee of the Bavarian Medical Association (Bayerische Landesärztekammer). All subjects gave written informed consent in accordance with the Declaration of Helsinki.

## Author Contributions

JL obtained funding, defined the study design, and oversaw quality control, analysis, interpretation of the data, and drafting of the report. PM analyzed the data and drafted the paper. NW, DZ, JS-M, and CJ were responsible for quality control and statistical analysis of the data. CM and ST were responsible for dietary intake assessment. SK, UN, and HB provided critical input on the application of the statistical methods. All authors read, critically commented on, and then approved the final manuscript.

### Conflict of Interest Statement

CJ was employed by company FGK Clinical Research GmbH, Munich. The remaining authors declare that the research was conducted in the absence of any commercial or financial relationships that could be construed as a potential conflict of interest.

## Abbreviations

24 h-DR: 24 h dietary recall
24HFL: 24-h food list
BVS II: the second Bavarian Food Consumption Survey
EFPQ: European Food Propensity Questionnaire
EPIC-Soft: European Prospective Investigation into Cancer and Nutrition-Software
EPIC: European Prospective Investigation into Cancer and Nutrition study
KORA: Cooperative Health Research in Augsburg
NCI: National Cancer Institute
MSM: Multiple Source Method
NVS II: the second National Food Consumption Survey.
